# A Mobile App (Concerto) to Empower Hospitalized Patients in a Swiss University Hospital: Development, Design, and Implementation Report

**DOI:** 10.2196/47914

**Published:** 2024-03-28

**Authors:** Damien Dietrich, Helena Bornet dit Vorgeat, Caroline Perrin Franck, Quentin Ligier

**Affiliations:** 1 Geneva Hub for Global Digital Health Faculty of Medicine University of Geneva Geneva Switzerland; 2 Kheops Technologies SA Plan-Les-Ouates Switzerland; 3 Geneva University Hospitals Geneva Switzerland

**Keywords:** patient empowerment, mobile apps, digital health, mobile health, implementation science, health care system, hospital information system, health promotion

## Abstract

**Background:**

Patient empowerment can be associated with better health outcomes, especially in the management of chronic diseases. Digital health has the potential to promote patient empowerment.

**Objective:**

Concerto is a mobile app designed to promote patient empowerment in an in-patient setting. This implementation report focuses on the lessons learned during its implementation.

**Methods:**

The app was conceptualized and prototyped during a hackathon. Concerto uses hospital information system (HIS) data to offer the following key functionalities: a care schedule, targeted medical information, practical information, information about the on-duty care team, and a medical round preparation module. Funding was obtained following a feasibility study, and the app was developed and implemented in four pilot divisions of a Swiss University Hospital using institution-owned tablets.

**Implementation (Results):**

The project lasted for 2 years with effective implementation in the four pilot divisions and was maintained within budget. The induced workload on caregivers impaired project sustainability and warranted a change in our implementation strategy. The presence of a killer function would have facilitated the deployment. Furthermore, our experience is in line with the well-accepted need for both high-quality user training and a suitable selection of superusers. Finally, by presenting HIS data directly to the patient, Concerto highlighted the data that are not fit for purpose and triggered data curation and standardization initiatives.

**Conclusions:**

This implementation report presents a real-world example of designing, developing, and implementing a patient-empowering mobile app in a university hospital in-patient setting with a particular focus on the lessons learned. One limitation of the study is the lack of definition of a “key success” indicator.

## Introduction

### Context

During recent decades, medicine has been moving from a focus on paternalistic approaches toward a paradigm of patient-centeredness, highlighting patient partnership and participation. Patient empowerment refers to a metaconcept with no unique definition [1]. However, it is commonly accepted that empowered patients possess key capacities and resources to be able to (1) participate in shared decision-making, (2) manage their own health, and (3) self-empower themselves [1].

### Patient Empowerment and Clinical Outcomes

Some studies have demonstrated a positive association between patient empowerment and improved clinical outcomes or their proxy. This is best documented in the context of chronic diseases, especially diabetes. Wong et al [2] compared serum glycated hemoglobin (HbA_1C_) and low-density lipoprotein cholesterol (LDL-C) levels in a group following implementation of a patient empowerment program (PEP) or the standard of care, resulting in decreased LDL-C levels in the PEP group. Similarly, Lian et al [3] found a lower incidence of all-cause mortality, cardiovascular events, and diabetes mellitus complications following participation in a PEP. In a review of randomized controlled trials, a decrease in HbA_1C_ and blood pressure levels was associated with empowerment interventions for patients with diabetes in sub-Saharan Africa [4]. In a meta-analysis, Baldoni et al [5] reported an improvement in HbA_1C_ levels following collective empowerment strategies. In a systematic review, Shnaigat et al [6] identified patient activation, a concept related to empowerment, as a valid strategy to improve outcomes of patients with chronic obstructive pulmonary disease.

However, it is important to highlight that several studies also reported no beneficial effects of empowerment programs. A 2017 meta-analysis found no statistically significant positive effect of empowerment on HbA_1C_ levels, despite five included studies reporting positive results [7]. Santoso et al [8] reported a lack of evidence to demonstrate a positive association between women’s empowerment and outcomes of child nutrition.

The lack of clear definitions and measures for empowerment may explain these controversial findings. Differences in program design could also contribute to this variability; therefore, further research identifying determinants for a successful intervention is needed. Indeed, the authors of the cited studies often reported the poor availability of high-quality research.

### Digital Health and Patient Empowerment

With the variety of solutions that could be envisioned, digital health is seen as a promising tool to promote patient empowerment and, indirectly, outcomes. However, mixed results are seen in the related literature.

In a systematic review, Johansson et al [9] showed that online communities support patient empowerment. Sosa et al [10] reported that a text messaging–based empowering intervention following head and neck surgery was both highly appreciated by patients and feasible. Conversely, Ammenwerth et al [11] reported no clinically relevant effect of patient portals on patient empowerment or health-related outcomes in a systematic review. Vitger et al [12] failed to demonstrate a positive effect of digital interventions to support shared decision-making, which was likely due to the small number of high-quality studies available. Verweel et al [13] found limited evidence demonstrating a positive effect of a digital intervention for health literacy. Finally, Thomas et al [14] reported that the quality and adequacy of the content of patient-empowering mobile apps varied greatly, urging for a more rigorous design and further testing before implementation. To our knowledge, no study has directly shown a link between mobile health app–induced empowerment and direct health outcomes.

Overall, few high-quality studies assessing the effect of digital health interventions on patient empowerment are available. Research is needed to confirm or deny the high perceived potential of digital tools.

### Concerto: A Mobile App Designed to Promote Patient Empowerment

Concerto is a mobile app designed to promote the empowerment of hospitalized patients. The app was initially designed during a hackathon in 2015 by a multidisciplinary team including health care and IT professionals as well as one patient. Building on the hackathon prototype and after a feasibility study, the Geneva University Hospitals (HUG) launched a project aiming at developing and implementing a fully functional mobile app delivered on institution-owned tablets in four pilot divisions (oncology, neurorehabilitation, orthopedics, and pediatrics) and assessing its effectiveness. Following this pilot study, the mobile app was further refined and deployed institution-wide based on a bring-your-own-device (BYOD) approach. This implementation report focuses on the pilot study only, with the objective to highlight the lessons learned. The report is structured following the iCHECK-DH (Guidelines and Checklist for the Reporting on Digital Health Implementations) guidelines [15].

## Methods

### Design and Agile Development

Building on the prototype developed during the hackathon, the foreseen functionalities of Concerto were first compared with patients’ expectations using focus groups and a semiquantitative questionnaire. A feasibility study was then performed to assess the availability and quality of the necessary data in the hospital health information system (HIS), which has been developed mainly in-house during the last 30 years.

Based on the patients’ insights, further described in Dietrich et al [16], version 1.0 of Concerto was specified and developed using an agile methodology with frequent user testing among hospitalized patients. The main functionalities of this version of Concerto included:

1. An up-to-date calendar on which patients can visualize their care schedule and better understand their daily planning with the aim to reduce the impact of these events and be better prepared for them.
2. A care team module on which the patient can obtain information about their on-duty care team, including names and photographs, to better know the professionals they will meet during their stay and facilitate communication.
3. A “questions” module on which patients can prepare their questions for health care professionals during the medical round and thus elevate the level of communication.
4. An “information” module on which patients have access to targeted medical information. Expected benefits of this module were to achieve better situation awareness, better treatment adherence, and early detection of complications.
5. A “practical information” module on which patients can find useful information about their stay at the hospital to improve their overall experience.
6. A social network module on which patients can interact with HUG accounts.

In subsequent versions, a new module was added, allowing the patient to choose their meal directly on the app rather than via a form completed by the nurse. This module was designed to simultaneously improve the patient experience while decreasing the nurse workload.

The app was developed in web languages (an Angular project), encapsulated as an iOS app (with Apache Cordova), and deployed on institution-owned tablets using a mobile device management solution. The key arguments for internal development over acquisition of a commercial solution were that (1) a significant part of the development work was about interfacing with the HIS, and (2) to our knowledge, no adequate and mature commercial solution was available at the time, although such solutions have emerged since then.

**Figure 1:**
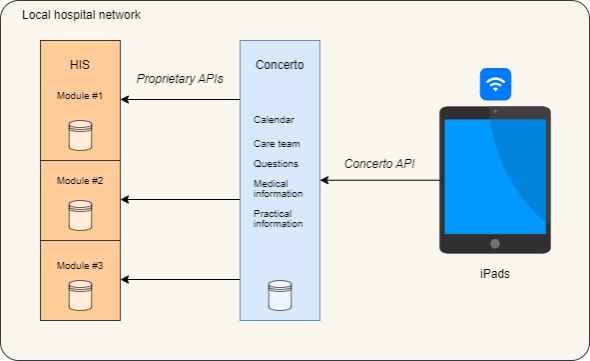
Technical overview of Concerto. Through the hospital's private Wi-Fi, the patient's tablet connects to the Concerto server, which contains some patient-generated data and the business logic to provide the app data. The server connects to different modules of the hospital information system (HIS) to retrieve other data using diverse application programming interfaces (APIs).

The initial version of the app connected directly to the custom-made HUG HIS using its proprietary interfaces for the sake of development simplicity and to alleviate time constraints. Further versions of the app have used industry standards such as Health Level 7 Fast Healthcare Interoperability Resources, with the vision to enable Concerto to connect more easily to other HISs in the future. This update has required new developments on the HIS side and was not achievable during the pilot phase described in this report. Figure 1 presents the simplified architecture of Concerto.

### Implementation

The definition of the logistics necessary to deliver Concerto on institution-managed tablets was an important part of the project. The following process was repeated for each patient: (1) setting up the tablet, including defining a personal passcode; (2) two-factor authentication in the Concerto app using the patient ID, scanned from the identification bracelet, and an SMS text messaging challenge; (3) on-demand charging; (4) disinfecting the tablet after the patient’s discharge; and (5) reinitializing and erasing the tablet. Tablets were charged and stored under key-secured storage in the nurse office. Each tablet was protected using individual cases. Hygiene procedures were validated by the Infection Prevention and Control Division of the HUG.

Once version 1.0 became available, caregivers of the different divisions were trained for 30 minutes during hands-on sessions in which (1) the project and app were presented; (2) the logistics of the tablets were explained; and (3) most importantly, they had the opportunity to familiarize themselves with the tool. At least one caregiver was defined as a “superuser” on a voluntary basis and was implicated from the beginning of the project. The specific responsibilities of superusers included (1) acquiring deep knowledge of the app, (2) being the focal point for exchange with the project team, and (3) acting as the referent for day-to-day questions of caregivers. A typical division included 20 beds and comprised a pool of over 50 caregivers that were trained during different sessions. Importantly, as in many hospital projects, caregivers did not have dedicated time for the project. Therefore, they had to manage making themselves available during a normal day of work.

One unit was scheduled for launch every 2 weeks, with constant presence of one member of the Concerto team during the first few days. Only patients able to interact with a mobile app, as assessed by their caregivers, were offered to use the app. To this end, caregivers used a communication flyer describing the functionalities of the app, the modalities of its use, as well as data and privacy considerations.

Bugs, feedback, and general satisfaction were systematically consigned to fuel the improvement-and-fix backlog.

### Data Considerations

At the stage presented during preparation of this report, Concerto worked mainly in “read-only” mode for personal health data available in the HIS and for insensitive, impersonal information. The information patients accessed from the HIS was part of their medical records. According to Swiss law, every patient owns the data contained in their medical record, except for personal notes of health care professionals, which were out of the scope of Concerto. Accordingly, Concerto facilitated access to data already owned by the patients.

The access to this sensitive personal information required a secure log-in based on the patient’s ID number and a second-factor authentication with an SMS text challenge. The use of institution-owned devices allowed Concerto to access data in the hospital’s local network, preventing unwanted access from the rest of the world.

The only personal information entered in Concerto included any questions patients may have had before interacting with their caregivers. This information was stored in the HIS and deleted after the hospital stay. Tablets were erased and reinitialized between patients, ensuring that no information leakage was possible between patients using the same tablet.

To summarize, Concerto facilitated the access to personal health information owned by the patient without the possibility to modify information from the app, and further allowed the patient to enter personal health information stored in the HIS that is inaccessible to others with all information systematically erased after the patient’s hospital stay.

Overall, the project was compliant with the Swiss Law for Data Protection [17].

### Funding and Budget Planning

The feasibility study and initial concept were self-funded by the eHealth and Telemedicine Division of the HUG, with the budget including salaries for a junior developer and a senior project manager.

The pilot project was then funded by private foundations based in Geneva, which included the salaries as well as necessary materials (tablets, covers, and software licenses).

Overall, the order of magnitude of the project costs ranged between US $150,000 and US $200,000, from which 25% was used for materials.

### Ethical Considerations

This study is based on an internal project of a Swiss University Hospital, aiming for quality improvement. As such, no patient or participant was included specifically for this study. Moreover, no patient data of any kind were collected. Accordingly, this study does not qualify for a review by the Geneva Canton Ethics Board (Commission Cantonale d’Éthique de la Recherche sur l’Être Humain [18]). As there were no participants involved in the research, no consent, compensation scheme, or privacy and confidentiality considerations applies.

## Implementation (Results)

### Project Summary

Concerto was implemented in four pilot divisions; a typical division includes 20 beds and comprises a pool of over 50 caregivers.

**Figure 2:**
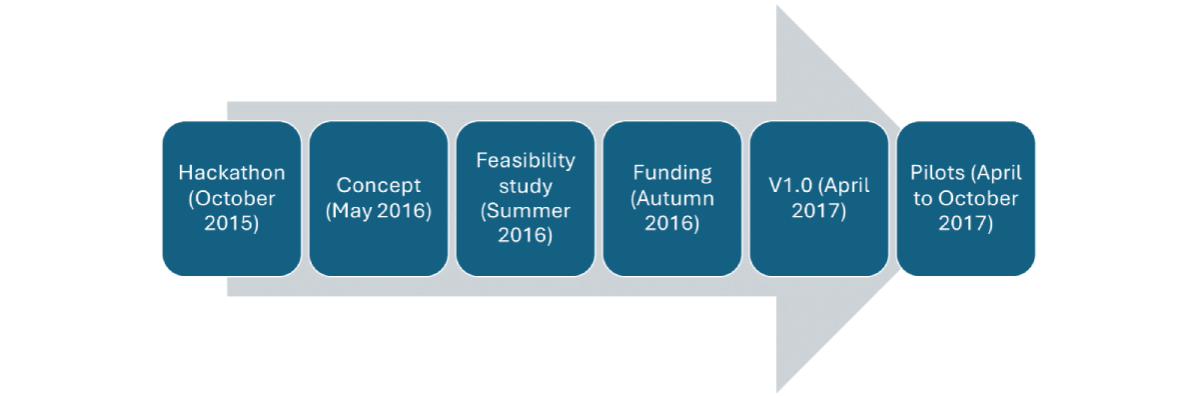
Timeline of the main phases of the project.

The timeline of the various stages of the project is provided in Figure 2. From the initial hackathon to acquiring the funding, approximately 1 year was necessary to refine the concept with patients and assess the feasibility of the app. Following funding acquisition, 6 months of development were needed, followed by 6 months of piloting in the four selected hospital divisions. Overall, the project took 2 years.

The budget was respected. However, additional funding would have been welcome to help free the caregivers from their clinical duties to enable better implementation (see below for further discussion of this point).

The development team considered the agile development phase to be efficient and productive.

Critical to the development process, the organization of focus groups and one-on-one interviews with patients were facilitated owing to the clinical background of the project manager. The development team reported that early contacts with the IT division during the feasibility study helped to improve communication and hence efficiency. Finally, dedicated support of the management unlocked political stalling.

During the pilot phase, the app was proposed to all eligible patients (see the Implementation subsection of the Methods). The percentage of eligible patients was unfortunately not systematically monitored but varied according to the profile of hospitalized patients across the different divisions and over time. An eligibility rate below 50% of all hospitalized patients was common.

The percentage of acceptance was also not systematically collected. However, the project team recalled acceptance to be relatively less variable than the percentage of eligible patients and consistently high (over 80%).

The dropout rate should have also been monitored carefully to identify the reasons for dropping out.

Most importantly, navigating the logistics of the tablet emerged as a particular challenge for caregivers. Despite the support of the project team, this impaired the inclusion of patients and consequently use of the app. More precisely, caregivers reported difficulties in assisting patients with the log-in and reinitialization procedures, and all logistics steps were reported as being too time-consuming. Based on this finding, it was decided to stop the pilot phase and transition to a BYOD approach.

### Lessons Learned and Determinants of Success and Failure

As often reported in the field, most challenges were encountered during the implementation (pilot) phase.

Our strategy to use institution-owned tablets added an important workload on the care teams because they were in charge of managing the tablet fleets in their divisions. This strategy was based on a rationale for cybersecurity and development; however, we underestimated the additional work it would generate for already overwhelmed caregivers. With such a strategy, it is our experience that protected and dedicated time for training caregivers is mandatory, at least for superusers. It is well recognized that the quality of training represents a key success factor for the implementation of electronic medical records (EMRs) [19]. Our impression is that this also applies to our project. Accordingly, our two first reported determinants of success are (1) having an implementation strategy that minimizes the impact on already overworked health care professionals and (2) including quality training time protected from the daily routine.

Similarly, we noticed that implementation was easier in care units in which the superuser was both convinced of the project’s benefits and was an influential figure among their peers. Accordingly, our third observed determinant of success is that the presence of a “killer function,” which on its own brings tremendous value, would have increased adoption by stakeholders. Even though such a function was not identified during patient focus groups, it was revealed during the implementation as the possibility for the patient to choose and order their own meals. Indeed, this function had the potential to both empower the patient and free up time for the caregivers. We consider that having such a functionality will be particularly relevant before the full-scale implementation.

The communication with the project’s stakeholders was considered to be a key factor to maintain motivation and trust in the project. In particular, reactivity in fixing identified bugs or transparency about delays was appreciated.

Finally, we realized that the quality of the HIS medical information fueling Concerto was not always appropriate for display in a patient mobile app. This was either because the information was not timely or was incorrect in some cases, but most importantly because its label was too technical. This issue was associated with disadvantages such as a lack of confidence in the project as well as advantages such as a welcome transparency about HIS data, triggering continuous improvements. For example, specific agenda labels designed for patients were created in the HIS owing to the implementation of Concerto.

## Discussion

This implementation report presents a real-world example of designing, developing, and implementing a patient-empowering mobile app in an in-patient setting of a Swiss public university hospital. The lessons learned, as presented in the Implementation (Results) section, are summarized in Table 1.

As described in the Introduction section, patient empowerment is a metaconcept. Hence, it is difficult to monitor with a single indicator. For this reason, a key success indicator was not defined at the beginning of this project, which has complicated its evaluation. This represents a limitation of this report, as an objective metric would have been important for complete evaluation. Simple monitoring metrics (eg, eligibility, number of users, and dropout and acceptance ratios) should have also been collected and are planned for the next app version. A randomized controlled trial assessing the effectiveness of the Concerto mobile app on a patient situation awareness score has been designed and should be conducted in the near future. This trial will allow for better evaluation of the cost-effectiveness of such a project. Overall, data on the effectiveness of eHealth projects are often lacking, and the creation of a dedicated “Implementation Report” article type in *JMIR Medical Informatics* is helping to fill this gap.

The generalizability of our study is another limitation. Indeed, the innovation ecosystem and the EMR landscape at the HUG are very specific and different constraints may be experienced in other settings. However, we believe the reported lessons learned remain relevant in various environments.

In response to one of the main lessons learned with the pilot implementation of Concerto, a BYOD version of the app was developed. With this version, every patient was able to use the app on their personal devices, including computers, tablets, or smartphones. This decision was made to limit the workload on caregivers and improve the adoption rate. New functionalities such as the possibility for patients to choose their meal were also developed to answer unmet needs for both end users and stakeholders impacted by implementation of the app (ie, caregivers). Important challenges in terms of cybersecurity, interoperability, and compatibility had to be met with development of the BYOD version. These will be further described in a forthcoming implementation report focusing on this project phase.

**Table 1:** Main lessons learned and associated perceived relevance.

Lessons learned	Perceived relevance^a^
Minimize the workload of caregivers or, if possible, decrease it	5/5
Plan protected time for training end users	4/5
Select a convinced and influential superuser	3/5
Wait for a killer function before implementing the app	5/5
Maintain trust through reactivity and transparent communication	4/5

^a^Based on perceived experience, lessons learned were identified by the authors and their relevance was assessed by consensus using a score ranging from 1 (minimally important) to 5 (maximally important).

## Abbreviations

BYOD: bring your own device
EMR: electronic medical record
HbA_1c_: glycated hemoglobin
HIS: health information system
HUG: Hôpitaux Universitaires de Genève (University of Geneva Hospitals)
iCHECK-DH: Guidelines and Checklist for the Reporting on Digital Health Implementations
LDL-C: low-density lipoprotein cholesterol
PEP: patient empowerment program
